# Dynamic Changes in Serum Glial Fibrillary Acidic Protein Levels Predicts Early Neurological Deterioration After Intravenous Thrombolysis in Minor Stroke

**DOI:** 10.1002/acn3.70552

**Published:** 2026-10-20

**Authors:** Jie Zhou, Yang Qu, Reziya Abuduxukuer, Peng Zhang, Ting Wang, Jian‐Hua Gao, De‐Wang Pan, Zhi‐Mei Yuan, Chun‐Ying Li, Li‐Ping Chen, Jin‐Hua Chen, Ji‐Liang Gu, An‐Ying Wang, Li‐Gang Jiang, Liang Liu, Yong‐Fei Jiang, Chun‐Fei Wang, Yan Jia, Hong Xin, Chun‐Li Jiang, Ying‐Bin Qi, Xue‐Feng Hu, Song Li, Hang Jin, Zhen‐Ni Guo, Yi Yang, the Biomarkers of Brain Injury Investigator Study Group, Lijuan Wang, Ting Wang, Jianhua Gao, Dewang Pan, Zhimei Yuan, Chunying Li, Liping Chen, Jinhua Chen, Jiliang Gu, Anying Wang, Ligang Jiang, Liang Liu, Yongfei Jiang, Chunfei Wang, Yan Jia, Hong Xin, Chunli Jiang, Yingbin Qi, Xuefeng Hu

**Affiliations:** ^1^ Stroke Center, Department of Neurology The First Hospital of Jilin University Changchun China; ^2^ Department of Neurology Songyuan Central Hospital Songyuan China; ^3^ Department of Neurology Jilin Neuropsychiatric Hospital Siping China; ^4^ Stroke Center, Department of Neurology Siping Central People's Hospital Siping China; ^5^ Department of Neurology Tonghua City Vascular Disease Hospital and Dongchang District People's Hospital Tonghua China; ^6^ Department of Neurology Songyuan Jilin Oilfield Hospital Songyuan China; ^7^ Stroke Center, Department of Neurology Dehuishi People's Hospital Changchun China; ^8^ Department of Neurology Affiliated Hospital of Jilin Medical College Jilin China; ^9^ Department of Neurology Songyuan Hospital of Integrated Traditional Chinese and Western Medicine Songyuan China; ^10^ Stroke Center, Department of Neurology Qianguoerros Mongolian Autonomous County Hospital Songyuan China; ^11^ Department of Neurology Jilin City Hospital of Chemical Industry Jilin China; ^12^ Department of Neurology Changchun People's Hospital Changchun China; ^13^ Department of Neurology Changchun Second Hospital Changchun China; ^14^ Department of Neurology Dongliao First People's Hospital Liaoyuan China; ^15^ Department of Neurology Jilin People's Hospital Jilin China; ^16^ Department of Neurology Jilin Province People's Hospital Changchun China; ^17^ Stroke Center, Department of Neurology Jilin Central General Hospital Jilin China; ^18^ Neuroscience Research Center, Department of Neurology The First Hospital of Jilin University Changchun China

**Keywords:** biomarkers, early neurological deterioration, glial fibrillary acidic protein, minor stroke

## Abstract

**Objective:**

Minor strokes may be associated with early neurological deterioration (END) and disabilities. The objective was to investigate the role of serum glial fibrillary acidic protein (GFAP) levels in predicting END.

**Methods:**

Data originated from a multicenter cohort study conducted prospectively. ΔGFAP was calculated as the ratio of post‐thrombolysis GFAP at 24 h to baseline GFAP. Univariable and multivariable binary logistic regression analyses were performed to assess the association between ∆GFAP and END, and a nomogram was established to illustrate its predictive value.

**Results:**

END occurred in 20 (7.35%) of the 272 patients with minor stroke who underwent intravenous thrombolysis (IVT). Elevated ∆GFAP was independently associated with END (OR, 3.50; 95% CI, 1.67 to 7.31; *p* < 0.001), even after adjusting for confounders (adjusted OR, 4.64; 95% CI, 1.86 to 11.54; *p* < 0.001). Furthermore, a model combining ∆GFAP, age, and National Institutes of Health Stroke Scale (NIHSS) score at admission demonstrated a good predictive value for END (AUC = 0.77; 95% CI, 0.67 to 0.89).

**Interpretation:**

∆GFAP is a strong, independent predictor of END in patients with minor strokes. Integrating ∆GFAP, age, and NIHSS score into a prognostic model results in a good discriminative ability for predicting END.

## 1 Introduction

Minor strokes, which are a category of ischemic strokes, involve mild neurological deficits and result in National Institutes of Health Stroke Scale (NIHSS) scores ranging from 0 to 5 [1, 2, 3]. In China, more than 50% of patients with ischemic strokes present with minor neurological deficits [4]. However, these minor strokes may not be clinically benign as early neurological deterioration (END) occurs in 2%–9% of these patients. Moreover, 10%–30% of patients with a minor stroke experience poor functional outcomes [5, 6, 7]. END is defined as an increase of ≥ 4 points on the NIHSS within 24 h [2, 3]. Therefore, investigating biomarkers to predict END in patients with minor strokes is essential.

Glial fibrillary acidic protein (GFAP) is an indirect fibrin present in the cytoskeleton and is vital for astrocyte maturation. It contributes to the structural support for neighboring cells and maintains the integrity of the blood–brain barrier [8, 9]. Clinical studies indicate that GFAP may be a promising biomarker for multiple sclerosis [10], stroke [11], and other neurological diseases, including Alzheimer's disease [12]. The levels of GFAP may differ between types and severity of strokes, and predict functional outcomes and cognitive dysfunction in patients with moderate‐to‐severe acute ischemic stroke [11, 13, 14]. In a previous study, we found that the serum levels of GFAP were correlated with the long‐term functional outcome of intravenous thrombolysis (IVT) [15]. Moreover, higher levels of GFAP are associated with astrocytic injury and blood–brain barrier dysfunction. Therefore, we aimed to investigate whether dynamic changes in patients with minor stroke treated with IVT are associated with END and to assess the association between ∆GFAP and END and develop a model including ∆GFAP for the prediction of END (≤ 24 h) after IVT.

## 2 Methods

### 2.1 Participants

This study used data from a prospective multicenter cohort study [15]. Post hoc analyses were performed on patients experiencing minor strokes who had sufficient serum sample volumes. From September 2016 to April 2023, patients receiving IVT were asked to consecutively enroll in the First Hospital of Jilin University. Additionally, patients were recruited from 15 hospitals located in Northeast China from September 2021 to January 2022 [15]. This study was approved by the Institutional Ethics Committee of the First Hospital of Jilin University (2015‐156). The inclusion criteria were as follows: (1) age > 18 years, (2) IVT administered within 4.5 h after symptom onset, (3) NIHSS score at admission ≤ 5, and (4) pre‐stroke Modified Rankin Scale (mRS) score of 0–1. Data analysis was performed on participants who met the inclusion criteria.

The study was granted by the Ethics Committee of the First Hospital of Jilin University (2015‐156) and followed the declaration of Helsinki. All patients gave written informed consent.

### 2.2 Clinical Parameters

Information on demographics, vascular risk factors, and clinical variables was collected at baseline. Risk factors for vascular events included cigarette smoking, alcohol consumption, hypertension, diabetes mellitus, dyslipidemia, prior ischemic stroke, coronary heart disease, and hyperhomocysteinemia [16, 17]. Stroke severity was evaluated at admission using the NIHSS score [18]. The Trial of Org 10172 in Acute Stroke Treatment (TOAST) classification was employed to classify the etiology of stroke [19]. The mRS score at 90 days was obtained through either in‐person or validated telephone‐based interviews. A favorable outcome was characterized by an mRS score of 0–1. END was defined as an increase in the NIHSS score ≥ 4 at 24 h after IVT [20]. Diffusion‐weighted magnetic resonance imaging was used to determine the infarct volume during hospitalization. The open source software 3D Slicer (version 5.3.0) [21] was used for image analysis. The NIHSS scores, infarct volumes, and 90‐day mRS scores were determined by independent assessors who were blinded to the clinical parameters and serum biomarker levels.

### 2.3 Blood Sampling and Serum GFAP Measurement

Blood was collected at baseline and 24 h after IVT administration. These samples were then centrifuged to obtain the serum and were subsequently transferred to cryogenic tubes and frozen at −80°C for future processing. The serum was stored in the Department of Biobank, Division of Clinical Research at the First Hospital of Jilin University until batch evaluation. The GFAP levels were determined using a magnetic particle‐based automated chemiluminescent enzyme‐linked immunosorbent assay system (MS‐Fast/Aceso 80A, Sophonix, Beijing, China) as indicated above [15]. This detection technology requires only 60–80 μL of serum and has a turnaround time of approximately 28 min from sample loading to result reporting. Serum samples were tested and analyzed by laboratory technicians at the Stroke Center of the First Hospital of Jilin University who were blinded to patients' clinical data. Test results were not shared with healthcare providers. The lower limit of quantification was 5 pg/mL, and the coefficient of variation was < 8.0%.

### 2.4 Statistical Analysis

Statistical analyses were conducted using SPSS software (version 27; IBM Corp, Armonk, NY, USA). Stata (version 15; Stata Corp LLC, College Station, TX, USA) and GraphPad Prism (version 10.1.2; GraphPad Software, San Diego, CA, USA) were utilized for data visualization. Categorical variables are expressed as frequencies and percentages, while group comparisons were performed using the *χ* ^2^ test or Fisher's exact test. GFAP change (∆GFAP) was defined as the ratio of the post‐thrombolysis GFAP level to the baseline level. Before statistical analyses, data were normalized using log_10_ transformation. Continuous variables that were normally distributed are expressed as mean ± SD, with group comparisons conducted using the *t*‐test; continuous variables that were not normally distributed are expressed as median ± IQR, with group comparisons conducted using the Mann–Whitney *U* test for two groups and the Kruskal–Wallis test or ANOVA for three groups.

Univariable and multivariable binary logistic regression analyses were performed to investigate the correlation between GFAP levels and END. In the study, four statistical models were used. Model 1 was unadjusted for any variable. Model 2 was adjusted for age and sex. Model 3 was further adjusted for risk factors such as cigarette smoking, alcohol consumption, hypertension, diabetes mellitus, dyslipidemia, prior ischemic stroke, coronary heart disease, and hyperhomocysteinemia. Finally, Model 4 was adjusted for admission blood pressure, admission heart rate, serum fasting glucose level, admission NIHSS score, onset‐to‐IVT time, ipsilateral vascular stenosis, and TOAST. These four models (Model 1–4) served exclusively for the purpose of independent association screening and were not intended for subsequent predictive performance comparison.

A logistic regression model was used to identify independent risk factors of END, which were defined as those that had a *p* value of ≤ 0.1, using backward multivariate logistic regression analysis. The risk factors identified were used to establish the nomogram. Two models were developed to assess the added predictive value of *∆*GFAP levels for END. Model A included only traditional clinical variables, whereas Model B, the final nomogram, incorporated both log_10_‐∆GFAP and selected clinical variables. The model was internally validated with a 10‐fold cross‐validation. Evaluation of models for discrimination ability was performed using receiver operating characteristic (ROC) curves and area under the ROC curve (AUC) values. In addition, the Hosmer–Lemeshow test, decision curve analysis (DCA), and nomograms were used to assess prognostic models. We not only performed a multivariable adjustment but also quantified the added predictive capabilities of ∆GFAP using several established conventional risk factors through the use of the AUC‐ROC, integrated discrimination improvement (IDI), and net reclassification index (NRI). A conventional statistical method [22] was employed to compare the two ROC curves. All tests were two‐sided, and *p* < 0.05 was considered statistically significant.

## 3 Results

### 3.1 Participant Characteristics

A total of 347 patients with minor stroke received IVT. After excluding 28 patients who did not receive the standard dose of alteplase or the bridging therapy, 319 patients met the inclusion criteria. Of these, two patients refused to participate, 39 had incomplete or unqualified blood samples, and six were lost to follow‐up at 90 days. This resulted in a final sample size of 272 that was used for analysis. The patient selection flowchart is shown in Figure S1.

Among the 272 patients, 20 (7.35%) experienced END. The characteristics of the patient populations in the END and NO‐END groups are summarized in Table S1. Among the demographics, vascular risk factors, and clinical data, intergroup differences were observed only in the admission NIHSS scores (3 vs. 4, *p* = 0.008). At the 90‐day follow‐up, 174 patients (64.0%) had favorable outcomes (Table 1). Patients in the highest ∆GFAP group had the lowest proportion of favorable outcomes (76.7% in T1 group vs. 63.0% in T2 group vs. 55.6% in T3 group, *p* = 0.016) (Table S2).

**TABLE 1:** GFAP levels and clinical outcomes between the END and NO‐END groups.

Variables	END (*n* = 20)	NO‐END (*n* = 252)	*X* ^*2*^/*Z*/*t*/*F*	*p*
Serum GFAP levels				
GFAP pre‐IVT (pg/mL)	10.25 (6.81–19.24)	8.54 (5.18–14.27)	−1.001	0.317
log_10_‐GFAP pre‐IVT (pg/mL)	1.04 ± 0.27	1.02 ± 0.35	0.125	0.724
GFAP post‐IVT (pg/mL)	40.05 (22.59–126.24)	13.57 (8.74–33.80)	−3.643	**< 0.001**
log_10_‐GFAP post‐IVT (pg/mL)	1.74 ± 0.64	1.29 ± 0.51	13.867	**< 0.001**
∆GFAP	3.57 (1.40–23.39)	1.57 (1.00–2.94)	−2.738	**0.006**
log_10_‐∆GFAP	0.70 ± 0.74	0.27 ± 0.47	13.584	**< 0.001**
Clinical outcomes				
Infarct volume (mL)^a^	1.14 (0.70–4.03)	1.32 (0.42–2.80)	−1.274	0.203
Favorable outcome, *n* (%)	5 (25.0%)	169 (67.1%)	14.224	**< 0.001**

*Note:* Significance of bold values indicate statistically significant results in *p* < 0.05.

Abbreviations: ∆GFAP, (GFAP post‐IVT)/(GFAP pre‐IVT); END, early neurological deterioration; IVT, intravenous thrombolysis; GFAP, glial fibrillary acidic protein; mRS, Modified Rankin Scale.

^a^ Infarct volume data were available for 249 patients because 23 patients were unable or unwilling to undergo MR examination (END group = 19, NO‐END group = 230).

### 3.2 Dynamic Serum GFAP Levels and END

Post‐IVT GFAP levels in the END group were significantly higher than those in the NO‐END group (40.05 vs. 13.57 pg/mL, *p* < 0.001) (Figure 1 and Table 1). A significantly greater increase in serum GFAP levels after IVT was observed in the END group (median ∆GFAP, 3.57‐fold) than that in the NO‐END group (median ∆GFAP, 1.57‐fold) (*p* = 0.006; Figure 1 and Table 1). Despite both patient groups having similar infarct volumes, the proportion of patients in the END group with a favorable prognosis at the 90‐day follow‐up was evidently lower than that in the NO‐END group (25.0% vs. 67.1%, *p* < 0.001) (Figure 2 and Table 1).

**FIGURE 1:**
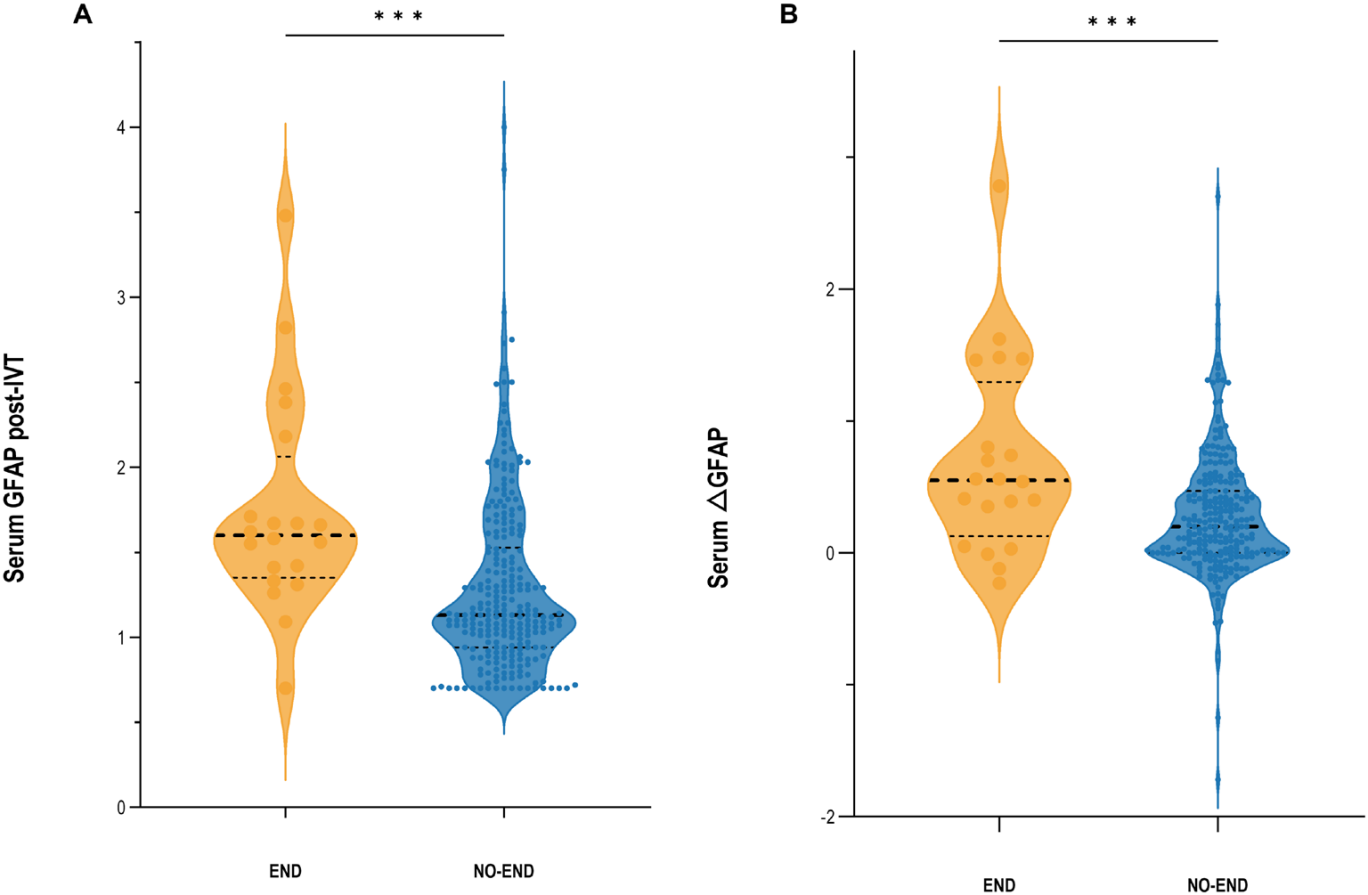
Comparison of serum GFAP levels between the END and NO‐END groups. (A) Serum GFAP levels post‐IVT between the two groups. (B) Serum ∆GFAP between the two groups. ∆GFAP, (GFAP post‐IVT)/(GFAP pre‐IVT); END, early neurological deterioration; GFAP, glial fibrillary acidic protein. The Serum GFAP levels post‐IVT and ∆GFAP underwent a base‐10 logarithmic transformation for statistical analyses. ***p < 0.001.

**FIGURE 2:**
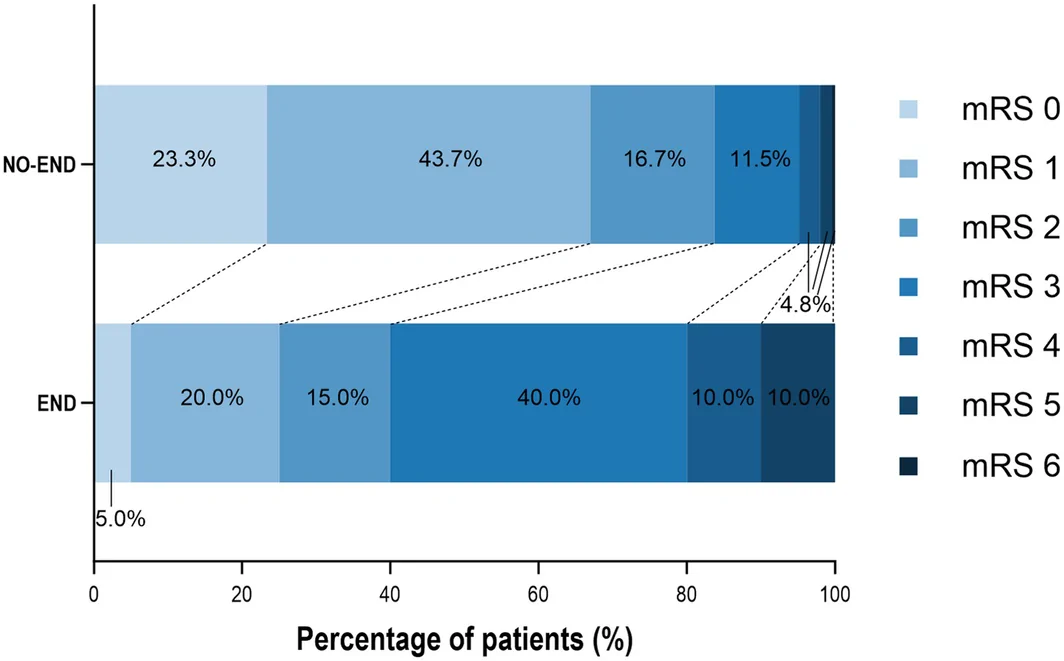
Comparison of 90d mRS between the END and NO‐END groups. END, early neurological deterioration; mRS, Modified Rankin Scale.

Both univariable and multivariable analyses revealed that ΔGFAP was an independent predictor of END (Figure 3 and Table S3). In the unadjusted model (Model 1), the OR was 3.495 with a CI of 1.672 to 7.306. This association remained statistically significant after adjusting for age, sex, vascular risk factors, and clinical factors across Models 2 to 4 (all *p* < 0.001). Notably, in Model 4, a 2.54‐fold (per log_10_‐∆GFAP) increase in ∆GFAP was associated with a 4.64‐fold increase in the risk of END (95% CI, 1.86 to 11.539; *p* < 0.001).

**FIGURE 3:**
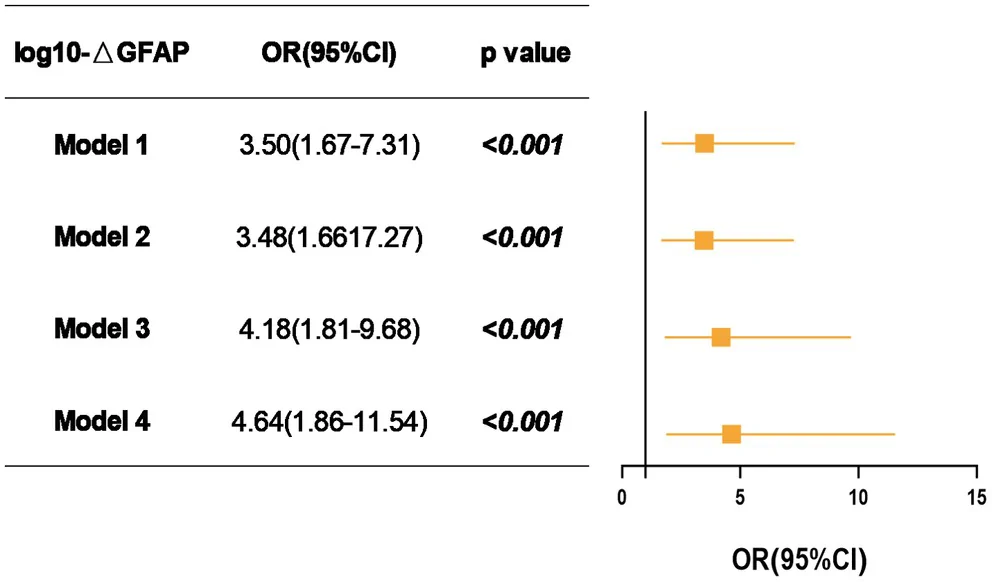
Multivariable binary logistic regression analyses between log_10_‐∆GFAP and END. Model 1 serves as the unadjusted baseline. Model 2 adjusts for age and sex, while Model 3 further incorporates risk factors such as cigarette smoking, alcohol consumption, hypertension, diabetes mellitus, dyslipidemia, previous ischemic stroke, coronary heart disease, and hyperhomocysteinemia, building upon the adjustments made in Model 2. Model 4 extends this analysis by including clinical data, specifically admission blood pressure, admission heart rate, serum fasting glucose, admission NIHSS score, onset‐to‐IVT time, ipsilateral vascular stenosis, and TOAST, in addition to all variables considered in Model 3. END was defined as an increase in NIHSS score ≥ 4 at 24 h after IVT. ∆GFAP, (GFAP post‐IVT)/(GFAP pre‐IVT); END, early neurological deterioration; GFAP, glial fibrillary acidic protein. Significance of bold values indicate statistically significant results in *p* < 0.05.

### 3.3 Construction of a Nomogram Prediction Model for END Risk

NIHSS score at admission and log_10_‐∆GFAP were additional independent predictors of END (Table S4). These variables were then integrated into the predictive model. Model A contained age and admission NIHSS scores. Model B included Model A and log_10_‐∆GFAP. The nomograms of the two models are shown in Figure 4. The AUC‐ROCs of the full and test models were similar (Model A: 0.70 vs. 0.61; Model B: 0.77 vs. 0.72), which suggested that END produced good stability (Figure 4 and Table 2). Further, the Hosmer–Lemeshow test indicated the model's good conformity (*p* > 0.05).

**FIGURE 4:**
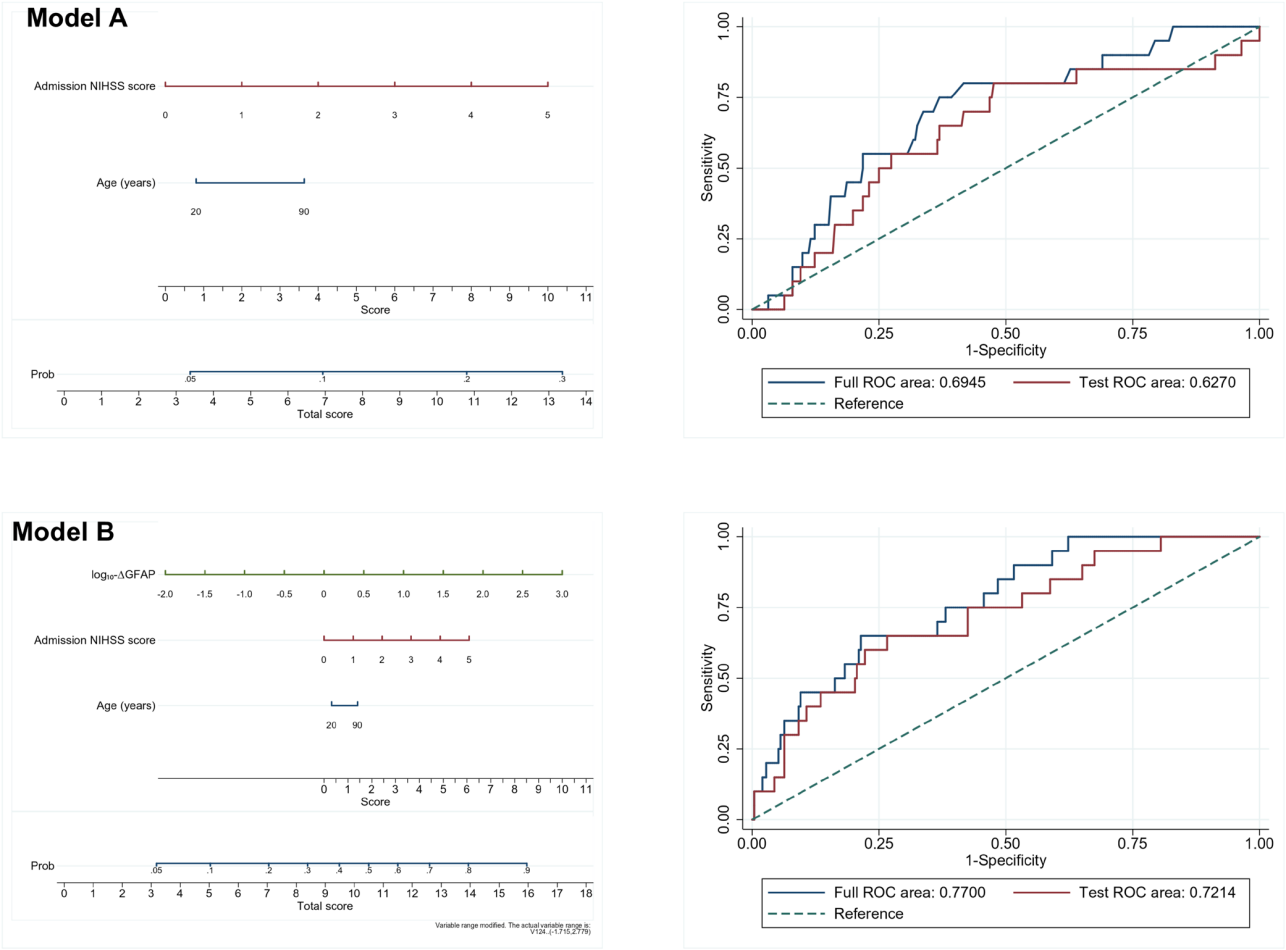
Nomogram models for predicting END. Nomograms (left), ROCs (right) of Model A and Model B. Model A included age and admission NIHSS score. Model B included Model A plus log_10_‐∆GFAP. ∆GFAP, (GFAP post‐IVT)/(GFAP pre‐IVT); GFAP, glial fibrillary acidic protein; NIHSS, National Institutes of Health Stroke Scale; ROC, receiver operating characteristic.

**TABLE 2:** Reclassification and discrimination statistics for END by ∆GFAP.

	AUC‐ROC	H–M		NRI			IDI		
	Estimate (95% CI)	*X* ^2^	*p*	Estimate (95% CI)	*Z*	*p*	Estimate (95% CI)	*Z*	*p*
Age, Admission NIHSS	0.695 (0.584–0.805)	7.713	0.462	Reference			Reference		
Age, Admission NIHSS, log_10_‐∆GFAP	0.770 (0.674–0.886)	4.852	0.773	70.00 (24.47–115.53)	3.013	**0.003**	6.62 (0.37–12.87)	2.070	**0.038**

*Note:* Significance of bold values indicate statistically significant results in *p* < 0.05.

Abbreviations: ∆GFAP, (GFAP post‐IVT)/(GFAP pre‐IVT); AUC‐ROC, the area under the receiver operating characteristic curve; CI, confidence interval; GFAP, glial fibrillary acidic protein; H–M, Hosmer–Lemeshow chi‐squared test; IDI, integrated discrimination improvement; NRI, net reclassification index; NIHSS, National Institutes of Health Stroke Scale.

There were no significant differences in the AUC‐ROCs for Model A and Model B. However, in Model B, the NRI (70.00%, *p* = 0.003) and IDI (6.62%, *p* = 0.038) increased significantly (Table 2). In the DCA, the higher curve indicated a greater net benefit across a given range of threshold probabilities; thus, the net benefit gained by adding ∆GFAP values (Model B) was greater than that in Model A (Figure 5).

**FIGURE 5:**
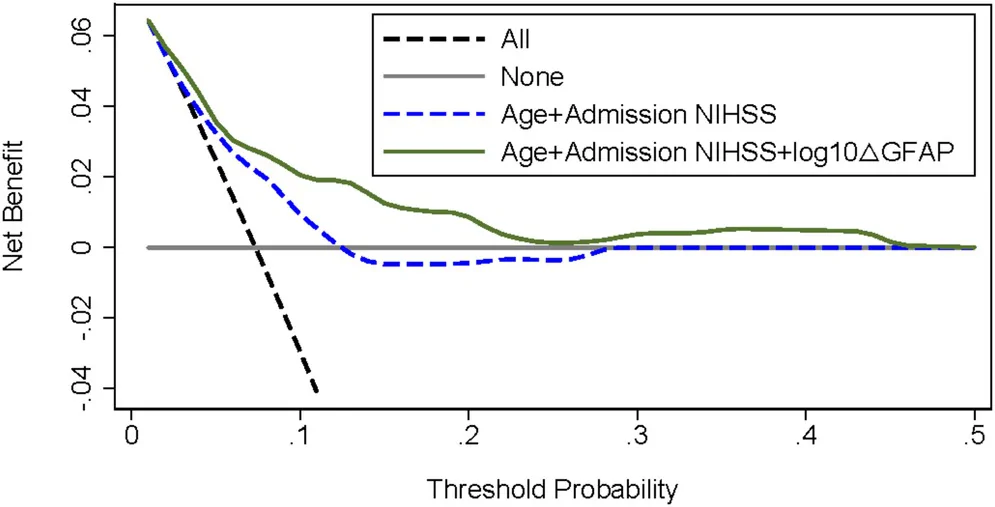
Decision curve analysis of the nomogram models. ∆GFAP, (GFAP post‐IVT)/(GFAP pre‐IVT); GFAP, glial fibrillary acidic protein; NIHSS, National Institutes of Health Stroke Scale.

## 4 Discussion

Among patients with a minor stroke receiving IVT, END was seen in 7.35% of patients, and END occurrence was associated with a poor prognosis at 90 days. Higher ∆GFAP levels were independent risk factors for END; a 2.54‐fold increase in ∆GFAP was associated with a 4.64‐fold increased risk for END. Thus, our results demonstrate the clinical utility of a ∆GFAP‐based nomogram approach and may function as a reliable estimate of END when combined with age and NIHSS score at admission (AUC = 0.770).

END has an unfavorable prognosis. Therefore, it is crucial to identify the potentially predictive biomarkers [20, 23, 24]. GFAP is a biomarker of acute central nervous system injury [10, 11, 12], and its usefulness has already been established. Interestingly, GFAP is also useful in the assessment of injury severity and prognostication in patients with acute ischemic stroke [25, 26]. Its sensitivity is particularly significant in minor strokes [13]. In patients with a minor stroke, serum GFAP levels were comparable before thrombolysis between the END and NO‐END groups. However, serum GFAP levels markedly increased after IVT in patients who experienced END. The longitudinal dynamic changes in GFAP, more than the single point measurement, were independently associated with END, reflecting the dynamic nature of clinical progression. Importantly, although GFAP levels can be influenced by age, stroke subtype, and etiology [11, 13, 27, 28], END and ∆GFAP maintained a statistically significant association even after adjusting for these variables. This supports the predictive power of ∆GFAP for END outcomes.

During the acute phase of AIS [9, 29], elevated GFAP mainly arises from two aspects: the extensive release of GFAP due to astrocyte necrosis within the infarct core, and the increased expression of GFAP associated with reactive gliosis in the surrounding area [30]. In our study, the infarct volume was comparable between the END and non‐END groups, indicating that GFAP from cell death in the infarct core likely doesn't differ much between them. This suggests that the differences observed are probably due to variations in reactive gliosis. Reactive gliosis is intricately linked to neuroinflammation, as the persistent overexpression of GFAP can induce the release of pro‐inflammatory cytokines and activate microglia, thereby intensifying neuroinflammatory processes and aggravating neuronal dysfunction. In conclusion, we propose that the observed differences in GFAP levels between the END and non‐END groups are more likely due to reactive proliferation rather than cell death within the core region. Furthermore, neuroinflammation, instigated by reactive proliferation, seems to be a fundamental mechanism underlying END.

Currently, the prediction of END in clinical practice mostly depends on traditional indicators such as age, admission systolic blood pressure, and NIHSS score [24, 31, 32]. These tools have clear limitations, particularly in patients with minor strokes who have NIHSS scores of 1–5. In our study, Model B showed a strong discrimination and stability, allowing for individualized risk estimation of END in the early post‐thrombolysis period. This model aided in early classification and enhanced supervision of high‐risk patients. Moreover, this model, which incorporated traditional risk factors in addition to the dynamic changes in the GFAP, was superior to Model A. The conversion to clinically applicable tools of statistical predictors increases their utility. Given that GFAP detection is reproducible, can be conducted at the bedside, and provides results within approximately 28 min, its integration into the predictive model would facilitate timely alerts for high‐risk patients and assist in identifying candidates for early neuroprotection and other targeted interventions.

GFAP is a promising candidate for stroke care and can be a potential biomarker of astroglial damage and activation. Additionally, GFAP has shown promise in prehospital diagnosis [27] and risk stratification of complications [15] as well as prognostication of clinical outcomes [25]. It has been increasingly used in prospective clinical trials as a biochemical endpoint [33]. Our results further support the potential of GFAP, which when combined with standard clinical parameters, can better predict END after IVT in patients with a minor stroke than any of the parameters alone. Nevertheless, external validation in multicenter large‐scale prospective cohorts is required to validate the generalizability of our findings. This model may help identify patients at high risk for targeted intervention. Future studies can assess whether such interventions will effectively reduce the incidence of END and ultimately improve long‐term outcomes in patients with a minor stroke.

Our study has several limitations. We did not differentiate between disabling and non‐disabling minor strokes. Further, we could not include a control group of patients receiving standard medical care without IVT. Finally, due to an insufficient sample size, we could not make more precise predictions for hemorrhagic and ischemic (END) conditions.

## 5 Conclusions

∆GFAP serves as a strong and independent predictor of END in patients with a minor stroke. Our predictive model, which combined ∆GFAP with age and NIHSS score at admission, provides good discriminative ability to predict END.

## Author Contributions

Each author participated in the data collection and analysis, as well as the writing and finalization of the manuscript. The corresponding author has access to all the data in this study and takes responsibility for the integrity of the research data and findings.

## Funding

This project was supported by the Science and Technology Department of Jilin Province (20250601002RC).

## Conflicts of Interest

The authors declare no conflicts of interest.

## Supporting information


**Figure S1:** Flowchart of the study. IVT, intravenous thrombolysis; NIHSS, National Institutes of Health Stroke Scale.
**Table S1:** Clinical characteristics between the END and NO‐END groups.
**Table S2:** Clinical characteristics and outcomes among tertiles of ∆GFAP.
**Table S3:** The association of ∆GFAP with END.
**Table S4:** Binary logistic regression analysis in END.

## Data Availability

The deidentified research data will be made available to qualified researchers upon reasonable request, allowing them to verify the experimental procedures and conclusions.

## Appendix 1 Coinvestigators

Lijuan Wang, Ting Wang, Jianhua Gao, Dewang Pan, Zhimei Yuan, Chunying Li, Liping Chen, Jinhua Chen, Jiliang Gu, Anying Wang, Ligang Jiang, Liang Liu, Yongfei Jiang, Chunfei Wang, Yan Jia, Hong Xin, Chunli Jiang, Yingbin Qi, Xuefeng Hu

**Table:**

Name	Location	Role	Contribution
Lijuan Wang	Department of Neurology, Songyuan Central Hospital, Songyuan, China	Member of the Biomarkers of Brain Injury Investigator Study Group	Acquisition of data
Ting Wang	Department of Neurology, Songyuan Central Hospital, Songyuan, China	Member of the Biomarkers of Brain Injury Investigator Study Group	Acquisition of data
Jianhua Gao	Department of Neurology, Jilin Neuropsychiatric Hospital, Siping, China	Member of the Biomarkers of Brain Injury Investigator Study Group	Acquisition of data
Dewang Pan	Stroke Center, Department of Neurology, Siping Central People's Hospital, Siping, China	Member of the Biomarkers of Brain Injury Investigator Study Group	Acquisition of data
Zhimei Yuan	Department of Neurology, Tonghua City Vascular Disease Hospital and Dongchang District People's Hospital, Tonghua, China	Member of the Biomarkers of Brain Injury Investigator Study Group	Acquisition of data
Chunying Li	Department of Neurology, Songyuan Jilin Oilfield Hospital, Songyuan, China	Member of the Biomarkers of Brain Injury Investigator Study Group	Acquisition of data
Liping Chen	Stroke Center, Department of Neurology, Dehuishi People's Hospital, Changchun, China	Member of the Biomarkers of Brain Injury Investigator Study Group	Acquisition of data
Jinhua Chen	Department of Neurology, Affiliated Hospital of Jilin Medical College, Jilin, China	Member of the Biomarkers of Brain Injury Investigator Study Group	Acquisition of data
Jiliang Gu	Department of Neurology, Songyuan Hospital of Integrated Traditional Chinese and Western Medicine, Songyuan, China	Member of the Biomarkers of Brain Injury Investigator Study Group	Acquisition of data
Anying Wang	Stroke Center, Department of Neurology, Qianguoerros Mongolian Autonomous County Hospital, Songyuan, China	Member of the Biomarkers of Brain Injury Investigator Study Group	Acquisition of data
Ligang Jiang	Department of Neurology, Affiliated Hospital of Jilin Medical College, Jilin, China	Member of the Biomarkers of Brain Injury Investigator Study Group	Acquisition of data
Liang Liu	Department of Neurology, Jilin City Hospital of Chemical Industry, Jilin, China	Member of the Biomarkers of Brain Injury Investigator Study Group	Acquisition of data
Yongfei Jiang	Department of Neurology, Changchun People's Hospital, Changchun, China	Member of the Biomarkers of Brain Injury Investigator Study Group	Acquisition of data
	Department of Neurology, Changchun Second Hospital, Changchun, China		
Chunfei Wang	Department of Neurology, Dongliao First People's Hospital, Liaoyuan, China	Member of the Biomarkers of Brain Injury Investigator Study Group	Acquisition of data
Yan Jia	Department of Neurology, Jilin People's Hospital, Jilin, China	Member of the Biomarkers of Brain Injury Investigator Study Group	Acquisition of data
Hong Xin	Department of Neurology, Jilin City Hospital of Chemical Industry, Jilin, China	Member of the Biomarkers of Brain Injury Investigator Study Group	Acquisition of data
Chunli Jiang	Department of Neurology, Jilin City Hospital of Chemical Industry, Jilin, China	Member of the Biomarkers of Brain Injury Investigator Study Group	Acquisition of data
Yingbin Qi	Department of Neurology, Jilin Province People's Hospital, Changchun, China	Member of the Biomarkers of Brain Injury Investigator Study Group	Acquisition of data
Xuefeng Hu	Stroke Center, Department of Neurology, Jilin Central General Hospital, Jilin, China	Member of the Biomarkers of Brain Injury Investigator Study Group	Acquisition of data
