# Supplementary material for: Dynamic Changes in Serum Glial Fibrillary Acidic Protein Levels Predicts Early Neurological Deterioration After Intravenous Thrombolysis in Minor Stroke
Source: Ann Clin Transl Neurol. 2026 Oct 20:10.1002/acn3.70552. Online ahead of print. doi: 10.1002/acn3.70552 (PMC13682409; doi:10.1002/acn3.70552)
Supplement: Supplementary file 1 [file ACN3-9999-0-s001.docx]

# Supporting Information

# Supplementary Figure S1. Flowchart of the study

Abbreviation: IVT = intravenous thrombolysis; NIHSS = National Institutes of Health Stroke Scale.

**Table S1. Clinical characteristics between the END and NO-END groups.**

| **Variables** | **END**  **（n=20）** | **NO-END**  **（n=252）** | ***X^2^/Z/t/F*** | **p** |
| --- | --- | --- | --- | --- |
| **Demographics** |  |  |  |  |
| Age (year) | 66±12.11 | 62.5±11.28 | 0.197 | 0.658 |
| Sex (male, n [%]) | 15(75.0%) | 194(77.0%) | 0.041 | 0.788 |
| **Vascular risk factors** |  |  |  |  |
| Cigarette smoking, n (%) | 13(65.0%) | 120(47.6%) | 2.240 | 0.165 |
| Alcohol consumption, n (%) | 8(40.0%) | 91(36.1%) | 0.121 | 0.810 |
| Hypertension, n (%) | 11(55.0%) | 142(56.3%) | 0.014 | 1.000 |
| Diabetes mellitus, n (%) | 4(20.0%) | 71(28.6%) | 0.676 | 0.605 |
| Dyslipidemia, n (%) | 6(30.0%) | 84(33.3%) | 0.093 | 1.000 |
| Previous ischemic stroke, n (%) | 2(10.0%) | 26(10.3%) | 0.002 | 1.000 |
| Coronary heart disease, n (%) | 5(25.0%) | 53(21.0%) | 0.174 | 0.776 |
| Hyperhomocysteinemia, n (%) | 8(40.0%) | 105(41.7%) | 0.021 | 1.000 |
| **Clinical data** |  |  |  |  |
| Admission SBP (mmHg) | 162.5±25.08 | 159±25.89 | 0.315 | 0.575 |
| Admission DBP (mmHg) | 95.5(83-100) | 90(80-101) | -0.745 | 0.456 |
| Admission heart rate (beats/min) | 78(68-80) | 79(70-89.75) | -1.415 | 0.157 |
| Serum fasting glucose (mmol/L) | 8.05(6.63-10.07) | 7.40(6.30-9.40) | -0.279 | 0.780 |
| Admission NIHSS score | 3(2-4) | 4(4-5) | -2.642 | ***0.008*** |
| Onset-to-IVT, min | 175(132-233.25) | 184.5(146-225) | -0.081 | 0.935 |
| TOAST, n (%) |  |  | 2.438 | 0.629 |
| LAA | 8(40.0%) | 63(25.0%) |  |  |
| SAO | 9(45.0%) | 139(55.2%) |  |  |
| CE | 1(5.0%) | 18(7.1%) |  |  |
| ODC | 0(0.0%) | 2(0.8%) |  |  |
| UE | 2(10.0%) | 30(11.9%) |  |  |
| Ipsilateral vascular stenosis, n (%) | 7(35.0%) | 55(22.2%) | 1.711 | 0.267 |

Notes:

*Ipsilateral vascular stenosis data were available for 268 patients because 4 patients were unable or unwilling to undergo vascular stenosis examination (END group=20, NO-END group=248).

Abbreviations: END = early neurological deterioration; IVT = intravenous thrombolysis; NIHSS = National Institutes of Health Stroke Scale; GFAP = glial fibrillary acidic protein; ∆GFAP = (GFAP post-IVT)/(GFAP pre-IVT). Significance of bold values indicate statistically significant results in p < 0.05.

**Table S2. Clinical characteristics and outcomes among tertiles of ∆GFAP**

| **Variables** | **Total（n=272）** | **T1**  **(∆GFAP≤1.00, n=73)** | **T2**  **(1.00＜∆GFAP ≤2.39, n=100)** | **T3**  **(∆GFAP＞2.39, n=99)** | ***X^2^/H/F*** | ***p*** |
| --- | --- | --- | --- | --- | --- | --- |
| **Demographics** |  |  |  |  |  |  |
| Age (year) | 63±11.32 | 61.58±12.90 | 62.18±10.62 | 62.18±10.86 | 0.076 | 0.927 |
| Sex (male, n [%]) | 209(76.8%) | 54(74.0%) | 75(75.0%) | 80(80.8%) | 1.403 | 0.496 |
| **Vascular risk factors** |  |  |  |  |  |  |
| Cigarette smoking, n (%) | 133(48.9%) | 36(49.3%) | 47(47.0%) | 50(50.5%) | 0.252 | 0.882 |
| Alcohol consumption, n (%) | 99(36.4%) | 28(38.4%) | 34(34.0%) | 37(37.4%) | 0.410 | 0.815 |
| Hypertension, n (%) | 153(56.3%) | 47(64.4%) | 50(50.0%) | 56(56.6%) | 3.554 | 0.169 |
| Diabetes mellitus, n (%) | 76(27.9%) | 18(24.7%) | 30(30.0%) | 28(28.3%) | 0.604 | 0.772 |
| Dyslipidemia, n (%) | 201(73.9%) | 52(71.2%) | 71(71.0%) | 78(78.8%) | 1.931 | 0.381 |
| Previous ischemic stroke, n (%) | 28(10.3%) | 10(13.7%) | 8(8.0%) | 10(10.1%) | 1.508 | 0.453 |
| Coronary heart disease, n (%) | 58(21.3%) | 17(23.3%) | 24(24.0%) | 17(17.2%) | 1.657 | 0.432 |
| Hyperhomocysteinemia, n (%) | 113(41.5%) | 29(39.7%) | 34(34.0%) | 50(50.5%) | 5.716 | 0.057 |
| **Clinical data** |  |  |  |  |  |  |
| Admission SBP (mmHg) | 160±25.80 | 159.07±26.67 | 158.97±26.44 | 161.30±24.66 | 0.253 | 0.777 |
| Admission DBP (mmHg) | 90(80-101) | 90(80-98.5) | 91(81-105) | 90(80-100) | 1.939 | 0.379 |
| Admission heart rate (beats/min) | 79(70-88) | 79(71-84) | 78(69.25-90) | 79(70-87) | 0.007 | 0.996 |
| Serum fasting glucose (mmol/L) | 7.5(6.3-9.4) | 7.8(6.5-9.83) | 7.1(6.2-8.6) | 7.8(6.7-10.2) | 6.000 | 0.050 |
| Admission NIHSS score | 4(3-5) | 4(3-4) | 4(3-5) | 4(4-5) | 3.002 | 0.223 |
| Onset-to-IVT, min | 183.5(144.5-225.0) | 184(152-231.5) | 190(140-224.75) | 178(142-225) | 0.887 | 0.642 |
| TOAST, n (%) |  |  |  |  | 10.768 | 0.170 |
| LAA | 71(26.1%) | 15(20.5%) | 20(20.0%) | 36(36.4%) |  |  |
| SAO | 148(54.4%) | 44(60.3%) | 59(59.0%) | 45(45.5%) |  |  |
| CE | 19(7.0%) | 4(5.5%) | 9(9.0%) | 6(6.1%) |  |  |
| ODC | 2(0.7%) | 1(1.4%) | 1(1.0%) | 0(0.0%) |  |  |
| UE | 32(11.8%) | 9(12.3%) | 11(11.0%) | 12(12.1%) |  |  |
| Ipsilateral vascular stenosis*, n (%) | 62(23.1%) | 7(9.7%) | 21(21.2%) | 34(35.1%) | 15.523 | ***<0.001*** |
| **Serum GFAP levels** |  |  |  |  |  |  |
| GFAP pre-IVT, pg/ml | 8.84(5.35-14.41) | 12.80(7.46-24.34) | 7.81(5.02-11.10) | 7.42(5.00-12.50) | 18.861 | ***<0.001*** |
| GFAP post-IVT, pg/ml | 14.15(9.15-40.90) | 8.70(5.12-14.32) | 11.79(8.57-15.70) | 45.44(21.14-105.34) | 123.747 | ***<0.001*** |
| ∆GFAP, pg/ml | 1.62(1.00-3.48) | 0.80(0.65-0.95) | 1.42(1.19-1.79) | 4.23(2.93-8.60) | 239.239 | ***<0.001*** |
| **Clinical outcomes** |  |  |  |  |  |  |
| END, n (%) | 20(7.4%) | 3(4.1%) | 3(3.0%) | 14(14.1%) | 9.525 | ***0.007*** |
| Infarct volume (ml)† | 1.31(0.46-2.89) | 0.96(0.30-2.76) | 1.27(0.31-2.43) | 1.81(0.58-3.36) | 5.492 | 0.064 |
| Favorable outcome, n (%) | 174(64.0%) | 56(76.7%) | 63(63.0%) | 55(55.6%) | 8.225 | ***0.016*** |

*Ipsilateral vascular stenosis data were available for 268 patients because 4 patients were unable or unwilling to undergo vascular stenosis examination (T1 group=72, T2 group=99, T3 group=97).

†Infarct volume data were available for 249 patients because 23 patients were unable or unwilling to undergo MR examination (T1 group=68, T2 group=86, T3 group=95).

Abbreviations: END = early neurological deterioration; IVT = intravenous thrombolysis; NIHSS = National Institutes of Health Stroke Scale; GFAP = glial fibrillary acidic protein; mRS = Modified Rankin Scale; ∆GFAP = (GFAP post-IVT)/(GFAP pre-IVT). Significance of bold values indicate statistically significant results in p < 0.05.

**Table S3. The association of ∆GFAP with END.**

|  | **Model 1** | | **Model 2** | | **Model 3** | | **Model 4** | |
| --- | --- | --- | --- | --- | --- | --- | --- | --- |
|  | ***OR*(95%CI)** | ***p*** | ***OR*(95%CI)** | ***p*** | ***OR*(95%CI)** | ***p*** | ***OR*(95%CI)** | ***p*** |
| log_10_-∆GFAP | 3.495  (1.672-7.306) | ***<0.001*** | 3.475  (1.661-7.268) | ***<0.001*** | 4.184  (1.808-9.681) | ***<0.001*** | 4.638  (1.864-11.539) | ***<0.001*** |

**Notes:**

Model 1 serves as the unadjusted baseline. Model 2 adjusts for age and sex, while Model 3 further incorporates risk factors such as cigarette smoking, alcohol consumption, hypertension, diabetes mellitus, dyslipidemia, previous ischemic stroke, coronary heart disease, and hyperhomocysteinemia, building upon the adjustments made in Model 2. Model 4 extends this analysis by including clinical data, specifically admission blood pressure, admission heart rate, serum fasting glucose, admission NIHSS score, onset-to-IVT time, ipsilateral vascular stenosis, and TOAST, in addition to all variables considered in Model 3. END was defined as an increase in NIHSS score ≥4 at 24 hours after IVT.

Abbreviations: END = early neurological deterioration; GFAP = glial fibrillary acidic protein; ∆GFAP = (GFAP post-IVT)/(GFAP pre-IVT). Significance of bold values indicate statistically significant results in p < 0.05.

**Table S4. Binary logistic regression analysis in END.**

| **Variables** | **Unadjusted** | | | **Adjusted** | | |
| --- | --- | --- | --- | --- | --- | --- |
|  | ***β*** | **OR(95%CI)** | ***p*** | ***β*** | **OR(95%CI)** | ***p*** |
| Admission NIHSS score | -0.421 | 0.657  (0.465-0.927) | ***0.017*** | -0.516 | 0.597  (0.413-0.862) | ***0.006*** |
| log_10_-∆GFAP | 1.251 | 3.495  (1.672-7.306) | ***<0.001*** | 1.347 | 3.848  (1.761-8.405) | ***<0.001*** |

Notes:

Abbreviations: NIHSS = National Institutes of Health Stroke Scale; GFAP = glial fibrillary acidic protein. ∆GFAP = (GFAP post-IVT)/(GFAP pre-IVT). Significance of bold values indicate statistically significant results in p < 0.05.
